# Evaluation of salt level and rigor status on the physicochemical and textural properties of low-fat pork sausages added with sea tangle extract using rapidly chilled pre-rigor pork ham

**DOI:** 10.5713/ab.23.0050

**Published:** 2023-05-04

**Authors:** Geon Ho Kim, Koo Bok Chin

**Affiliations:** 1 Department of Animal Science, Chonnam National University, Gwangju 61186, Korea

**Keywords:** Pork Sausage, Pre-rigor Muscle, Rapid Chilling, Reduced-salt, Sea Tangle Extract

## Abstract

**Objective:**

This study was performed to evaluate the quality characteristics of pork sausage (PS) with sea tangle extract (STE) and rapid chilled pre-rigor muscle (RCPM) for the development of reduced-salt low-fat sausage.

**Methods:**

Pre- and post-rigor pork ham muscles were prepared to process PSs. Positive control (reference, REF) using post-rigor muscle were manufactured at a regular-salt level of 1.5%. Fresh and rapid-chilled pre-rigor muscle (FPM and RCPM) were used to manufacture reduced-salt sausages with 0.8% salt. Reduced-salt PSs were prepared with four treatments: FT1 (FPM alone), FT2 (FPM with 5% STE), RT1 (RCPM alone), and RT2 (RCPM with 5% STE). The physicochemical and textural properties of the sausages with reduced-salt levels and RCPM combination were measured to determine if the characteristics of RCPM were similar to those with FPM.

**Results:**

The pH values of PS with FPM and RCPM were higher than those of REF with post-rigor muscle. Color values (L*, a*, b*) were not affected by different rigor-states and salt addition level. Textural properties of reduced-salt PSs were similar to those of REF due to the improved functionalities of pre-rigor muscle. RT2 had lower expressible moisture (%) than other treatments with post-rigor muscle and RCPM except for RT1.

**Conclusion:**

The addition of STE and RCPM to reduced-salt PS increased the water-holding capacity, which was lower than those of PS with STE using RCPM but similar to those of regular-salt sausage.

## INTRODUCTION

The addition of salt (NaCl) is important for the functional properties of meat products. Salt add flavor and taste to food. It is also added to food to prevent the growth of pathogens by lowering water activity [1]. It helps improve the quality characteristics of meat products by extracting salt-soluble protein that can affect meat products functionalities such as emulsification, texture properties, water-holding capacity (WHC), and protein gelation [2].

Salt plays an important role in the human body and health by regulating blood pressure, water content, and electrolyte balance, and maintaining plasma volume [3]. However, excessive salt intake causes damage to the cardiovascular system by increasing blood pressure. It causes conduit artery narrowing, resulting in hypertension, which is a fatal harmful condition that causes cardiovascular diseases such as stroke and coronary heart diseases [4]. Although the World Health Organization recommends a salt intake of ≤5 g/d, many people consume salt in foods more than twice the recommended amount of salt [5]. Thus, effective strategy of reducing salt intake should be developed.

Hot boning is a fabrication method that involves taking lean meat and fat from carcass in a pre-rigor state before chilling [6]. Pre-rigor muscle produced through hot boning can reduce the cost of chilling and storage and increase the final yield of meat product manufacturing [7]. Pre-rigor muscle tissues have improved cooking yield, WHC, and emulsifying property as compared to post-rigor one based on their higher pH [8,9]. Therefore, pre-rigor muscles may be appropriate for manufacturing reduced-salt meat products because of their better processing properties with superior the technological functional properties than those of post-rigor muscles. After death, the pH values of beef and pork muscles decrease as the ability to retain water decreases [10]. Therefore, a method to prevent the decrease in the pH of pre-rigor muscle during manufacturing of meat products should be applied.

Rapid chilling methods can be used to maintain the meat quality of pre-rigor muscle. Lee et al [11] demonstrated that gel forming ability and processing aptitude improve when crust-frozen air chilling is applied to hot-boning pork ham muscle. Neto et al [12] reported that the pH of *M. longissimus lumborum* of rapid-chilled beef does not change during 4 h of chilling. Tomović et al [13] also showed that the pH of rapid chilled (−31°C) *M. semimembranosus* of pork carcass and conventionally air-chilled (2°C to 4°C) one does not differ 8 h post-mortem. The rate of pH decline is slower, and a higher pH is desirable for manufacturing cooked cured products. Liu et al [14] reported that the rapid chilling (−20°C) of pork *M. longissimus dorsi* can delay the rate of pH with an accelerating temperature decline and has a higher solubility of sarcoplasmic and myofibrillar proteins and purge loss than conventional chilling. Thus, rapid chilling is suitable for maintaining the high pH of pre-rigor muscle with great processing aptitude.

Sea tangle (*Laminaria japonica*) is brown algae that grows in East Asian seaside; it contains high concentration of glutamic acid and aspartic acid, which are ingredients that can enhance food flavor [15]. Alginate, a water-soluble dietary fiber in seaweeds such as sea tangle, improves the WHC and antimicrobial activity of meat products [16]. Kim et al [17] demonstrated that sea tangle powder increases the WHC, texture, and flavor properties of breakfast sausage. Choi et al [18] reported that the addition of sea tangle powder to reduced-fat pork patty could improve quality characteristics similar to those of regular-fat one. In addition, treatments added with l% or 3% sea tangle powder have higher scores of juiciness and overall acceptability than the control without this powder in sensory evaluation using a 10-point descriptive scale by twelve panelists. López-López et al [19] performed sensory evaluation and showed that low-fat frankfurters added with 5% edible seaweed had more desirable flavor and texture. Seaweeds, including sea tangle, are abundant sources of bioactive compounds such as phenolic compounds, carotenoid pigments, and polysaccharides [20]. Since they have antioxidant and antimicrobial activities, they can preserve the sensory properties and shelf-life of foods by retarding oxidation and microbial spoilage [21]. Therefore, sea tangle may be an effective functional ingredient for improving the functional and sensory properties of reduced-salt meat products.

Since salt reduction may decrease the functional properties of meat products, strategies that can replace the essential function of salt should be developed to manufacture reduced-salt meat products. The combination of pre-rigor muscle and sea tangle extract (STE) could be a potential for salt reduction, and rapid chilling might maintain the muscle characteristics of pre-rigor muscle. Therefore, this study was conducted to evaluate the quality characteristics of low-fat pork sausages added with STE by using rapid-chilled pre-rigor raw meat for the development of reduced-salt meat products.

## MATERIALS AND METHODS

### Materials

Raw pork at two rigor-states were purchased from a local meat market (Hyundai distribution, Gwangju, Korea). Pork hams were prepared with post-rigor muscle obtained more than 24 h after slaughter and pre-rigor muscle obtained less than 1 h after slaughter (delivery in with insulation for 30 min). Then, external fat and connective tissues were removed and chopped with a meat chopper (M-12S; Hankook Fujee Industries Co., Ltd., Hwaseong, Korea). The pre-rigor muscle was rapidly chilled in a freezer (DS-301; Unique Daeseung Co., Ltd, Pocheon, Korea) at −30°C until the internal temperature reached approximately 0°C for 40 min. STE was prepared by heating dried sea tangle with water at a ratio of 1:9 at 90°C for 2 h. After heating, mixture was filtered with a testing sieve (AST 50 300 μm; ChungGye Industrial Mfg., Co, Seoul, Korea) and stored in refrigerator at 4°C until used in manufacturing sausages.

### Experimental design

The formulation of pork sausages is shown in Table 1. For comparison with reduced-salt sausages with 0.8% salt using fresh or rapid-chilled pre-rigor muscle, a positive control (REF) with 1.5% salt was set using post-rigor muscle. Thus, five treatments were prepared: REF with 1.5% salt using post-rigor pork ham, FT1 with 0.8% salt using fresh pre-rigor pork ham, FT2 with 0.8% salt and 5% STE using fresh pre-rigor pork ham, RT1 with 0.8% salt using rapid-chilled pre-rigor pork ham, and RT2 with 0.8% salt and 5% STE using rapid-chilled pre-rigor pork ham.

### Preparation of pork sausages

Pork sausages were prepared based on 2 kg per treatment. Raw meat, soy protein isolate (fat replacer), and ice water were ground with a bowl cutter (K-15; Talsa, Valencia, Spain) for 30 s. During FT2 and RT2 manufacturing, ice water and STE were added. Curing agents such as salt, sodium erythorbate, sodium nitrite, and sodium polyphosphate were added to meat batters and mixed with ice water for 1 min. Then, flavoring agents such sugar, corn syrup, and spices and the remaining ice water were added and ground twice for 1 min. The sausage batters were stuffed into a polyvinylidene chloride casing, and heated in a water bath (WB-22; Daihan Scientific Co., Ltd., Seoul, Korea) at 75°C for 30 min until the internal temperature reached 72°C. After being heated, pork sausages were chilled in an ice box for 30 min. The whole parameters of cooked sausages were measured immediately after cooling was complete.

### Determination of the pH and temperature of pre- and post-rigor raw pork ham

For the measurement of parameters that indicate the rigor state of muscles, the pH values and temperatures of raw pork ham were measured. The pH values of raw meat and cooked sausages were measured with an electrode-type pH meter (Model 340; Mettler-Toledo, Greifeense, Switzerland). The temperature of raw meat was measured with a thermometer (52II; FLUKE, Everett, WA, USA). Results were expressed as the means of five measurement times.

### Color values of cooked sausages

The following color values of pork sausages were measured six times by using colorimeter (CR-10; Minolta Co., Ltd., Osaka, Japan): lightness (Commission Internationale de l’Eclairage [CIE] L*), redness (CIE a*), and yellowness (CIE b*). The means and standard deviation of the values measured six times were calculated to derive the results. Those of a white flat plate were measured and standardized (CIE L* = 94.8, CIE a* = 1.0, CIE b* = 0.1) to check the consistency of the CIE values.

### Salinity

The salinity of the sausages was measured as follows. After the mixture of homogenized sausage (5 g) and distilled water (20 mL) was heated at 90°C for 30 min, the sample was centrifuged at 1,660×g for 15 min (Supra 22; Hanil Scientific Inc., Kimpo, Korea). Then, the salinity of the supernatant was determined with a salinity electrode-type meter (TM-30D; Takemura Electric Works, Ltd., Tokyo, Japan).

### Expressible moisture and cooking loss

A 1.5 g cuboidal sample was wrapped with three-quartered filter papers (Whatman #3; GE Healthcare, Little Chalfont, UK), placed in a 50 mL conical tube (SPL Life Science Co., Ltd., Pocheon, Korea), and centrifuged at 1,660×g for 15 min.


$$\text{Expressible moisutre }(\text{EM},\%)=\frac{Expressible\;water\;weight\;of\;filter\;paper}{Sample\;weight}\times 100$$

Cooking loss (CL, %) was derived by substituting the weight of the sample before and after heating in the following formula:


$$\text{Cooking loss }(\text{CL},\%)=\frac{\left(Sample\;weight\;before\;cooking-Sample\;weight\;after\;cooking\right)}{Sample\;weight\;before\;cooking}\times 100$$

### Texture profile analysis

Samples with a diameter of 1.25 cm and a height of 1.3 were prepared, and the hardness (gf), springiness (mm), gumminess, chewiness, and cohesiveness were measured 10 times by using an Instron Universal Machine (Model 3344; Instron, Norwood, MA, USA). A 500 N load cell was used to compress the samples twice to 75% of the height of the initial samples height at a speed of 300 mm/min.

### Statistical analysis

The preparation of pork sausages and the whole experiments were performed in triplicate. Results were expressed as mean and standard deviation in IBM SPSS Statistics (Ver. 26.0 for Windows; IBM, Chicago, IL, USA). Data were subjected to one-way analysis of variance (ANOVA) by using the difference between treatment groups as a factor, and the post-ANOVA was conducted at a 95% significant level via Duncan’s multiple range test.

## RESULTS AND DISCUSSION

### pH and temperature values of raw pre-rigor muscle

Table 2 shows the pH values of raw meat as affected by different rigor states. The pH values of post-rigor ham were lower than those of fresh and rapid-chilled pre-rigor ham (p<0.05). Conversely, the pH values did not differ between fresh and rapid-chilled pre-rigor ham (p>0.05). The pH values of post-rigor muscle declined rapidly during refrigerated storage for 24 h after slaughter, whereas pH values of pre-rigor muscle were higher than that of post-rigor muscle due to without chilling. Kim and Chin [22] reported that the pH values of pre-rigor muscle (pH 6.06) were higher than that of post-rigor muscle (pH 5.69). This finding was similar to the present results. The pH of raw meat is an important factor affecting the WHC of the final processed meat. As pH increases from the isoelectric point, the binding of myofibrillar proteins strengthens, thereby increasing the WHC of muscle proteins [23]. Thus, fresh and rapid-chilled pre-rigor muscle with a high pH could retain a great processing aptitude of meat products.

The temperature of raw meat differed among all states (Table 2). Specifically, the temperatures of rapid-chilled pre-rigor ham were the lower than those of other raw meats (p< 0.05). Van Laack et al [24] observed that the pH and temperature values of hot-processed *M. triceps brachii* (pre-rigor) were higher than those of cold-processed muscle (post-rigor) regardless of phosphate addition. Since the temperature of fresh pre-rigor ham was measured immediately after slaughter without undergoing a cooling process, it was close to the body temperature of carcass before slaughter. Since the temperature of post-rigor ham was close to the refrigerated storage temperature at 10°C, while the temperature of rapid-chilled pre-rigor ham was approximately 0°C, the temperature difference between the two meat states was significant.

### pH values of cooked sausages

Table 3 shows the pH values of pork sausages with the different salt levels and rigor state of raw meat. REF, which was manufactured with post-rigor raw meat and 1.5% salt, had lower pH values than other treatments (p<0.05), indicating that the pH values of pork sausages with pre-rigor muscle might be slightly higher than those of pork sausages with post-rigor muscle. The pH values of cooked sausages differed due to the pH value of raw meat, itself. Motycka and Bechtel [8] demonstrated that pH values of pre-rigor muscle were higher than those of post-rigor muscle in pork *M. biceps femoris* before cooking. pH values varied even after cooking, possibly contributing to an increase in cooking yield. Neto et al [12] reported that the pH values of beef pre-rigor *M. Longissimus lumburum* didn’t change during rapid chilling for 4 h, and cooking loss didn’t increase during 14 days of storage. Therefore, rapid chilling might not affect the pH values of cooked pork sausages and could be used for the storage and distribution of pre-rigor raw meat without changing functional properties such as WHC.

### Color values of cooked sausages

The color values of cooked pork sausages are shown in Table 3. Lightness (CIE L*), redness (CIE a*), and yellowness (CIE b*) did not differ among all treatment (p>0.05). Therefore, the different rigor states of raw meat and the addition of STE did not affect the color values of pork sausages. Kim and Chin [22] reported that the lightness values of pork sausage added with 1.5% salt were lower than those with 0.5% salt. In a previous study, the salt content of pork sausages manufactured with post-rigor meat at 1.5% salt was three times higher than those with pre-rigor muscle at 0.5%. The reason for the change in lightness of sausages might to be related to water release during heating processing. However, pre-rigor muscle and STE which can increase WHC of meat product [8,18] were used to manufacture reduced-salt sausages in this study, and therefore increased water retention would not change in lightness of reduced-salt sausages in this study. Cooking loss of meat products decrease with increasing salt addition level [2]. Fernández-López et al [25] reported that the addition of salt to minced meat can increase WHC during manufacturing process, resulting in reduction of lightness values and reflection. Villamonte et al [26] found no differences in lightness and redness between pork meat batters with 1.5% and 3% salt under the same phosphate addition level. These results indicated that the salt level of meat products did not influence color under all conditions and might not be affected depending on the range of differences in the salt addition level. Van Laack et al [24] evaluated the sensory properties of cooked cured pork shoulder and ham and obtained similar color scores in both parts regardless of the rigor state of raw meat. As a result, salt addition level and differences in rigor state did not affect the color of cooked pork sausages in this study.

### Salinity of cooked sausages

Table 3 presents the results of the salinity of pork sausages. The salinity values of REF were the highest among all treatments (p<0.05). The salt addition level (1.5%) in REF had similar salinity values of cooked sausages, and these values were higher than those of other treatments with 0.8% salt. No differences in salinity values were observed among all treatments except for REF because of the same salt addition level (p>0.05). The addition of STE did not affect pH values. Choi et al [27] reported that the salinity of frankfurter with 1.5% NaCl was higher than that with 1.0% NaCl, and the addition of sea tangle powder (1.0%) increases the salinity of frankfurter by 0.21%. Despite the addition of STE (5%), the salinity values of RT1 and FT1 were not different from those of RT2 and FT2 in this study. These results indicated that the effect of STE on salinity was minimal.

### Expressible moisture (%)

The expressible moisture (EM) percentage of pork sausages is shown in Table 4. The EM percentage of REF with the higher salt content (1.5%) was similar to those of FT1 and RT1 (p>0.05) and higher than those of FT2 and RT2 (p<0.05). The results indicated that the WHC) of pre-rigor processed treatments could reduce the added salt level. In general, meat products contain high sodium or salt content that improves their WHC. Puolanne et al [28] reported that raw batter and cooked sausages with high salt contents (2.5%) had an increased WHC compared with sausages with low salt contents (1.5%). However, the trend of the WHC in the present study was different from that in their study; that is, FT2 and RT2 with reduced-salt level (0.8%) had a higher WHC than REF with a high salt level (1.5%) in this study. Motycka and Bechtel [8] showed that the WHC of pre-rigor pork ham was higher than that of post-rigor ham based on the higher pH values of pre-rigor muscle than that of post rigor muscle. In addition, the EM percentages of FT2 and RT2 were lower than those of RT1, indicating that the addition of STE might improve the WHC of cooked pork sausage regardless of the rigor state of raw meat. Choi et al [18] reported that the WHC of low-sodium frankfurter added with sea tangle and sea mustard was improved. The dietary fiber of seaweeds had higher WHC and viscosity by forming gel. Although the rapid chilling did not affect the EM percentage as compared to the treatments with the same addition levels of salt and STE, the chilling rate was highly associated with retaining pH and WHC of muscles. Raw meat chilling can improve the WHC with reducing postmortem temperature by prevention shrinkage and denaturation of myofibrillar protein due to fast decrease in pH [29]. In addition, the pH decline of postmortem muscle is affected by the chilling rate, and rapid chilling can decrease the drip loss of pork meat by avoiding pre-rigor shortening, which affects WHC with contraction of muscle fiber during postmortem [30]. Therefore, using pre-rigor pork ham and STE for reduced-salt pork sausages could decrease the EM percentage and improve the WHC, and rapid chilling might retain processing properties such as the WHC of pre-rigor raw meat.

### Cooking loss (%)

Cooking loss (CL, %) did not differ among all treatments (p>0.05). Salt increases the hydration and water-binding capacity of protein, thereby improving protein binding properties and cooking loss [2]. As salt contents increase, CLs decrease. However, no differences in CL between REF and reduced-salt treatments were observed in this study (p>0.05). The meat products manufactured with pre-rigor muscle had a higher cooking yield (CY) [7]. No effects of the addition of STE or rapid-chilling were observed on CLs of reduced-salt pork sausages with pre-rigor muscle. Lee et al [11] prepared pork sausage from pre-rigor muscle subjected to crust-frozen air chilling in a freezer at −29°C until the internal muscle temperature reached −4°C; they found that the pre-rigor muscle has a higher CY than the post-rigor muscle. These results indicated that rapid chilling could maintain the better functionality of pre-rigor muscle under conditions similar to those set in this study. Therefore, reduced-sodium sausages using pre-rigor pork ham could have CL similar to those of REF; as a result, pre-rigor pork ham could improve the water-binding capacity of pork sausages regardless of the rapid chilling rate of pre-rigor muscle.

### Texture profile analysis

The textural properties of all treatments were similar (p>0.05; Table 4). The hardness, springiness, gumminess, chewiness, and cohesiveness values of reduced-salt treatments were not different from those of regular-salt control (REF). Salt-soluble protein extracted by salt addition could contribute to the formation of textural properties, and the gel forming system of myofibrillar proteins is composed of protein and salt. Thus, salt level might considerably affect the textural properties of meat products. However, differences in textural properties among the different salt contents were not observed in this study, and it differed from the general trend of texture profile analysis as affected by salt reduction. Dowierciał et al [31] showed that the texture properties and rating of canned ham manufactured with pre-rigor pork were higher than those of post-rigor processed ham. In the present study, pre-rigor muscle combined with reduced-salt treatments could be used as a supplement to reduce salt. Furthermore, pre-rigor raw meat, which has a higher binding ability than post-rigor raw meat, can improve the textural properties of reduced-salt meat products. The addition of STE to pork sausages did not affect the textural properties in this study. However, this effect of sea tangle was not observed in the present study because of the addition of different densities and extraction methods of sea tangle. Kim and Chin [22] observed that pork sausage manufactured with pre-rigor pork ham stored in a freezer at −30°C for 4 wks had lower hardness and gumminess values than pork sausage with fresh pre-rigor muscle. The rapid chilling of pre-rigor raw meat did not affect the textural properties of sausages in the present study, indicating that the textural properties differed between the two studies. Muscle freezing can influence the rheological characteristics of meat products [32]. However, in this study, rapid chilling did not cause the severe denaturation of myofibrillar protein during rapid freezing, and the protein structure might be consistent [11]. Therefore, rapid-chilled pre-rigor muscle rather than regular-chilled muscle could be used to manufacture pork sausage with enhanced functionalities.

## CONCLUSION

The EM percentages of rapid chilled pre-rigor pork sausages added with STE (RT2) were lower than those of treatments using pre-rigor muscle without STE (RT1) and REF. Color, CL, and textural properties did not differ among all treatments. The use of pre-rigor muscle combined with the addition of STE improved the functional properties of reduced-salt meat products. In addition, the processing properties of pre-rigor processed raw meat were retained by rapidly chilling raw meat. In conclusion, the use of pre-rigor muscle and 5% STE in pork sausages could reduce the amount of added salt from 1.5% (regular salt level) to 0.8%. The addition of STE improved the WHC of reduced-salt pork sausages. Therefore, the rapid chilling of pre-rigor raw meat combined with STE could be applied to manufacture reduced-salt meat products without any defects.

## Figures and Tables

**Table 1:** Formulation of low-fat pork sausages with different salt and rigor state

Ingredients (%)	Treatments^1)^				
	REF	FT1	FT2	RT1	RT2
Pork ham	60.0	60.0	60.0	60.0	60.0
Water	33.23	33.23	28.23	33.23	28.23
Non meat ingredients					
Sodium chloride	1.27	0.57	0.57	0.57	0.57
Sodium tripolyphosphate	0.40	0.40	0.40	0.40	0.40
Sodium erythorbate	0.05	0.05	0.05	0.05	0.05
Cure blend^2)^	0.25	0.25	0.25	0.25	0.25
Sugar	1.00	1.00	1.00	1.00	1.00
Spices	1.00	1.00	1.00	1.00	1.00
Fat replacer					
Soy protein isolate	1.50	1.50	1.50	1.50	1.50
Konjac and Carrageenan	1.00	1.00	1.00	1.00	1.00
Whole-fat milk powder	1.00	1.00	1.00	1.00	1.00
Sea tangle extract	0.00	0.00	5.00	0.00	5.00
Total	100.7	100.0	100.0	100.0	100.0

1) REF, Pork sausage (PS) with 1.5% of salt using raw post-rigor muscle; FT1, PS with 0.8% of salt using fresh pre-rigor muscle; FT2, PS with 0.8% of salt and 5% of sea tangle extract using fresh pre-rigor muscle; RT1, PS with 0.8% of salt using rapid chilled pre-rigor muscle; RT2, PS with 0.8% of salt and 5% of sea tangle extract using rapid chilled pre-rigor muscle

2) Composed of 93.75% salt and 6.25% sodium nitrite.

**Table 2:** pH and temperature values of raw meat at different rigor-states

Items	Post-rigor ham	Fresh pre-rigor ham	Rapid chilled pre-rigor ham
pH	5.62±0.16^b^	6.27±0.03^a^	6.18±0.04^a^
Temperature (°C)	10.2±0.42^b^	30.3±2.24^a^	0.35±0.21^c^

a–c Means having the same superscripts in the same row are not significantly different (p>0.05).

**Table 3:** pH, color values, and salinity of low-fat cooked pork sausages

Parameters	Treatments^1)^				

	REF	FT1	FT2	RT1	RT2
pH	6.00±0.00^b^	6.24±0.02^a^	6.23±0.04^a^	6.23±0.02^a^	6.18±0.04^a^
CIE L*	65.1±1.23^NS^^2)^	64.2±2.35	64.2±2.53	66.7±4.31	67.5±2.61
CIE a*	8.97±0.16^NS^	10.7±0.74	9.79±2.13	9.50±1.65	9.69±1.23
CIE b*	10.6±0.13^NS^	10.1±0.95	10.9±0.57	11.2±1.43	11.9±0.04
Salinity (%)	1.52±0.14^a^	0.78±0.05^b^	0.80±0.03^b^	0.77±0.07^b^	0.82±0.05^b^

CIE, Commission Internationale de l’Eclairage.

1) REF, pork sausage (PS) with 1.5% of salt using raw post-rigor muscle; FT1, PS with 0.8% of salt using fresh pre-rigor muscle; FT2, PS with 0.8% of salt and 5% of sea tangle extract using fresh pre-rigor muscle; RT1, PS with 0.8% of salt using rapid chilled pre-rigor muscle; RT2, PS with 0.8% of salt and 5% of sea tangle extract using rapid chilled pre-rigor muscle.

2) NS, not significant.

a,b Means having the same superscripts in the same row are not significantly different (p>0.05).

**Table 4:** Expressible moisture, cooking loss, and textural properties of low-fat cooked pork sausages

Parameters	Treatments^1)^				

	REF	FT1	FT2	RT1	RT2
Expressible moisture (%)	28.5±1.08^a^	27.6±0.67^ab^	25.9±0.42^b^	28.1±0.73^a^	26.3±0.69^b^
Cooking loss (%)	1.47±0.01^NS^^2)^	1.48±0.07	1.60±0.21	1.64±0.25	1.52±0.03
Hardness (gf)	2,741±33.9^NS^	2,730±220	2,852±218	2,667±197	2,622±375
Springiness (mm)	4.73±0.42^NS^	4.34±0.52	4.00±0.37	4.34±0.22	4.15±0.45
Gumminess	23.9±1.41^NS^	24.1±2.69	23.8±4.53	25.7±2.83	24.5±4.17
Chewiness	101±12.0^NS^	91.3±16.5	92.9±11.5	93.8±30.8	93.2±23.8
Cohesiveness	0.01±0.00^NS^	0.01±0.00	0.01±0.00	0.01±0.00	0.01±0.00

1) REF, pork sausage (PS) with 1.5% of salt using raw post-rigor muscle; FT1, PS with 0.8% of salt using fresh pre-rigor muscle; FT2, PS with 0.8% of salt and 5% of sea tangle extract using fresh pre-rigor muscle; RT1, PS with 0.8% of salt using rapid chilled pre-rigor muscle; RT2, PS with 0.8% of salt and 5% of sea tangle extract using rapid chilled pre-rigor muscle.

2) NS, not significant.

a,b Means having the same superscripts in the same row are not significantly different (p>0.05).
